# A *Dictyostelium discoideum* mitochondrial fluorescent tagging vector that does not affect respiratory function

**DOI:** 10.1016/j.bbrep.2020.100751

**Published:** 2020-03-25

**Authors:** Christopher J. Perry, Eleanor C. Warren, Joseph L. Damstra-Oddy, Claire Storey, Lisa M. Francione, Sarah J. Annesley, Paul R. Fisher, Annette Müller-Taubenberger, Robin S.B. Williams

**Affiliations:** a Centre for Biomedical Sciences, School of Biological Sciences, Royal Holloway University of London, Egham, Surrey, TW20 0EX, UK; b Department of Physiology, Anatomy and Microbiology, La Trobe University, Melbourne, Victoria, 3086, Australia; c Department of Cell Biology, Biomedical Center, LMU Munich, 82152, Planegg-Martinsried, Germany

**Keywords:** *Dictyostelium discoideum*, Mitochondria, Fluorescent tagging, Live cell imaging, MLS, mitochondrial localization sequence, REMIT, red mitochondrial-tagging plasmid, RFP, red fluorescent protein, ROS, reactive oxygen species, TOM/TIM, Translocase of the outer/inner membrane

## Abstract

Visualizing mitochondria in living *Dictyostelium discoideum* cells using fluorescent dyes is often problematic due to variability in staining, metabolism of the dyes, and unknown potential effects of the dyes on mitochondrial function. We show that fluorescent labelling of mitochondria, using an N-terminal mitochondrial localization sequence derived from the *D. discoideum* protein GcvH1 (glycine cleavage system H1) attached to a red fluorescent protein enables clear mitochondrial imaging. We also show that this labelling has no effect upon mitochondria load or respiratory function.

## Highlights

• Live-cell visualization of mitochondria dynamics is currently difficult in *Dictyostelium discoideum*.
• We provide an easy and rapid procedure for visualizing mitochondria.
• This technology employs a plasmid, REMIT-mRFPmars, expressing a mitochondrial targeting sequence linked to mRFPmars.
• Expression of REMIT-mRFPmars does not alter mitochondrial load or function, providing a suitable tag for live-cell studies.

## 1 Introduction

Mitochondria play important roles, most notably cellular energy production by oxidative phosphorylation [1], hence the aptly coined phrase “the powerhouse of the cell” [2]. Mitochondria are also involved in Ca^2+^ management [3], production of ROS [4], redox signalling [5,6] and apoptosis [7]. Many studies investigating mitochondrial function observe mitochondrial morphology and their dynamics within the cell [8]. Such observations can be achieved by the use of various fluorescent dyes such as Rhodamine 123 (R123) [9], tetramethylrhodamine-methyl-ester (TMRM) [10] and JC-1 (tetraethylbenzimi-dazolylcarbocyanine iodide) [11]. However, these dyes rely upon a mitochondrial membrane potential and can be washed out if the mitochondria experience depolarisation [12]. Furthermore, these dyes are unsuitable for use with aldehyde fixation due to resulting changes in mitochondrial metabolic state [12]. Other fluorescent dyes developed for visualizing mitochondria include the Mitotracker Red and Green dyes. Mitotracker Red binding depends on both the presence of a mitochondrial membrane potential while Mitotracker Green binding does not. These dyes can be used in combination with a number of cell fixation methods, however, they may cause cytotoxic effects following prolonged use. Other methods of real time mitochondrial imaging include the use of fluorescently tagged mitochondrial localised proteins, where the tagged protein is recognised by the mitochondrial ‘Translocase of the outer/inner membrane’ (TOM/TIM) protein complexes and transported into the mitochondria [13]. The transport of mitochondrial proteins into the mitochondrial matrix is facilitated by an N-terminal pre-sequence [14]. This pre-sequence can consist of 10–80 amino acid residues and is usually cleaved off by the matrix processing peptidase following localization [15]. However, these fluorescent proteins can interfere with the function of the native protein and impede mitochondrial function. As such, non-cytotoxic mitochondrial markers are required.

*Dictyostelium discoideum* is a tractable model system widely used for research in a range of fields including cell and developmental biology, evolutionary biology, as well as in immunology and molecular pharmacology studies. In cell and developmental biology, *D. discoideum* is often used to improve our understanding of cell motility [16,17]. In molecular pharmacology research, *D. discoideum* has been used to investigate mechanisms of action of pharmaceutical drugs including treatments for epilepsy [[18], [19], [20]] and neurodegenerative disorders [21,22]. Other studies investigate mechanisms of action of bioactive natural products such as curcumin, naringenin and a range of bitter tastants [[23], [24], [25]]. One recent study, identifying a mitochondrial protein, GcvH1, involved in the cellular function of cannabinoids on the glycine cleavage system [26], highlights the presence of an N-terminal mitochondrial localization sequence and thus raises the possibility of using this sequence for mitochondrial tagging in *D. discoideum*. To further these studies, a non-cytotoxic mitochondrial marker is required that can be used in *D. discoideum* without affecting cellular respiratory function.

In this study we created a novel expression plasmid (REMIT; red mitochondria) for real time visualization of mitochondria in *D. discoideum*. The REMIT plasmid allows the expression of an enhanced RFP protein (mRFPmars) [27] with a mitochondrial localization sequence situated at its N-terminus. We show that transfection of REMIT into *D. discoideum* cells facilitates the localization of mRFPmars to the mitochondrial matrix. We also show that the presence of mRFPmars within the mitochondria has no effect on mitophagy or cellular respiratory function. We therefore present a method that allows the real time visualization of mitochondria within *D. discoideum* cells with no deleterious effects.

## 2 Methods

### 2.1 Creation of the REMIT plasmid and over-expression *D. discoideum* cell lines

**Fig. 1:**
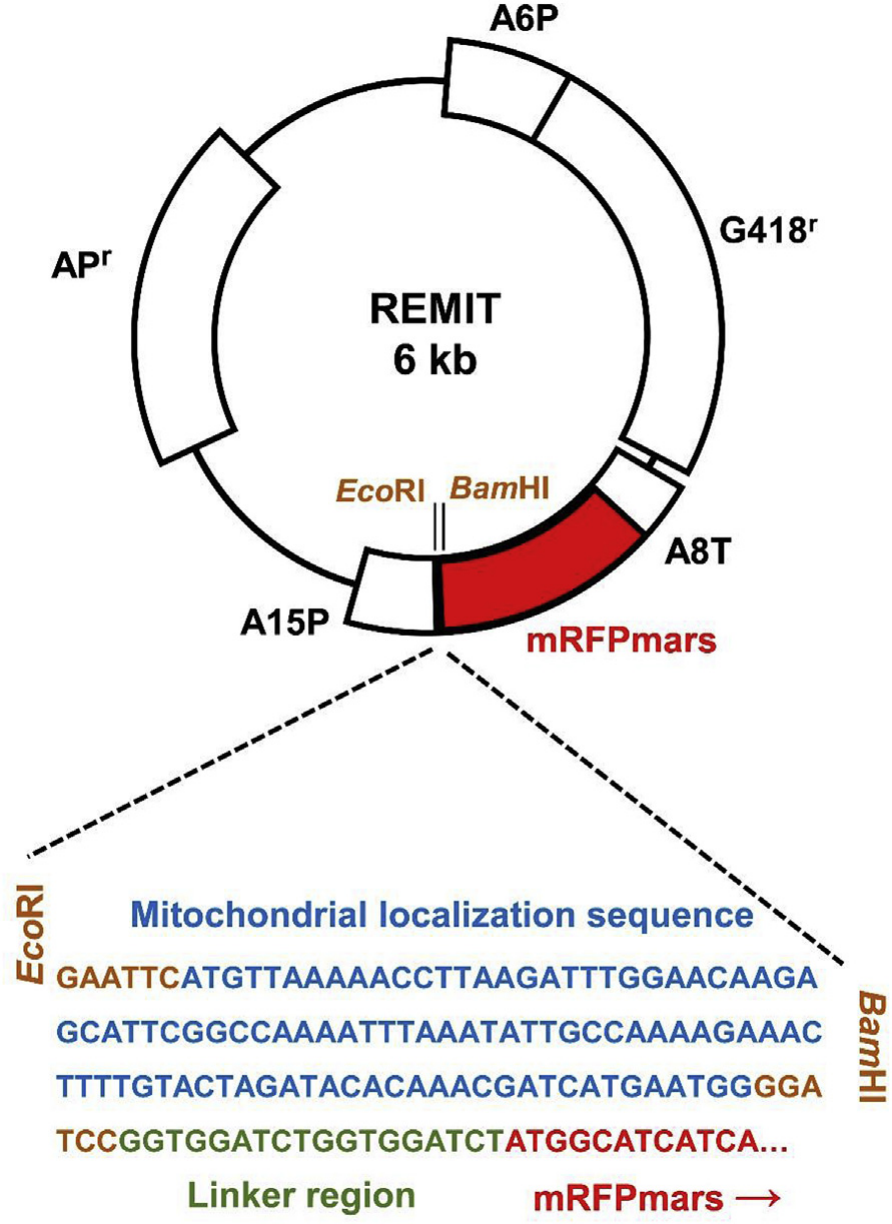
**REMIT plasmid construction**. The mitochondrial localization sequence was ligated into the pDXA-389-2 plasmid 5’ of the mRFPmars gene using *Eco*RI and *Bam*HI restriction sites. Actin 6 promoter, A6P; actin promoter 15, AP15; Ampicillin resistance cassette, AP^r^; monomeric red fluorescence protein, mRFPmars; actin terminator 8, A8T; Geneticin resistance cassette, G418^r^. (For interpretation of the references to colour in this figure legend, the reader is referred to the Web version of this article.)

Primers (fwd: ATAGAATTCATGTTAAAAACCTTAAGATTTG and rev: TATGGATCCCCATTCATGATCG) complementary to the 5’ region of *gcvH1* were used to amplify the 99-bp mitochondrial localization sequence (Fig. 1). The PCR product was digested with *Eco*RI and *Bam*HI and cloned into the extra chromosomal plasmid pDXA-389-2 [27,28]. The PCR product was inserted into the multiple cloning site located immediately 5’ of the mRFPmars gene, and sequenced to confirm that no mutations were introduced. Insertion of the PCR product at this location enabled expression of a 27 kDa mRFPmars with a 33 amino acid residue localization sequence linked to its N-terminus via a 6 amino acid residue linker region (Fig. 1). The resulting plasmid was transfected into wildtype (AX3) cells and selected using geneticin (10 μg/ml) to create a REMIT over-expression cell line [29]. The GREMIT plasmid was based upon the same targeting region inserted into the GFP vector pDM1209 [30].

### 2.2 Fluorescence and live-cell microscopy

For immunolabeling, cells expressing REMIT were plated on round 12-mm glass coverslips, and after 20 min were fixed with 15% picric acid/2% paraformaldehyde in 10 mM PIPES, pH 6.0, for 20 min and post-fixed with 70% ethanol for 10 min [31]. Cells were then washed three times in PBS, once with 10 mM PIPES, and twice with PBS/1% glycine, and incubated in blocking buffer (PBS plus 2% bovine serum albumin) for 1 h at room temperature (RT). After blocking, the cells were washed three times with PBS and incubated with primary antibodies (2 μg/ml mouse monoclonal anti-porin antibody (Developmental studies hybridoma bank (DSHB); 70-100-1) [32], and 1:1000 rat anti-RFP (6G6 anti-red rat mAb, Chromatek) for 2 h, followed by the incubation with secondary antibodies (1:1000 Alexa 488-conjugated goat anti-mouse IgG (Invitrogen; A28175) and 1:1000 rabbit anti-rat (Alexa fluor® 488 rabbit anti-rat IgG, Life technologies), for 1 h. After immunostaining, samples were washed three times in PBS and embedded using Fluoromount-G^TM^, with DAPI (1:1000 of 1 mg/ml DAPI dissolved in methanol; Invitrogen, 00-4959) to stain DNA. For live-cell microscopy, cells were seeded in μ-dishes (Ibidi, 80606), or open chambers as described previously [28].

Confocal microscopy was performed at the Bioimaging core facility of the Biomedical Center (LMU Munich) using an inverted Leica TCS SP8 equipped with lasers for 405, 488, 552, and 638 nm excitation. Images were acquired with a HC PL APO 63x/1.40 oil PH3 objective. Recording was sequential to avoid bleed-through. Alexa-488, and RFP were recorded with the hybrid photo detectors, DAPI with the conventional photomultiplier tube.

High resolution live-cell imaging was performed using an inverted Zeiss LSM 900 Airyscan 2 microscope [33]. Images were acquired with a Plan-Apochromat 63x/1.40 oil DIC objective with a GaASP-PMT detector (450–700 nm) in the MPCX SR-4y modus at an excitation of 558 nm. Z-stacks (25 corresponding to 4.32 μm) were recorded over time (2.55 s per z-stack). 3D reconstructions of single z-stacks were performed using the Imaris software package (Bitplane, Zurich, Switzerland).

### 2.3 Western blot analysis to monitor mitochondrial loading

Cell lysates (30 μg) were separated by gel electrophoresis, transferred to nitrocellulose membranes (Merck Millipore, IPFL00010), and analysed by Western blotting. A mouse anti-porin primary antibody (0.2 μg/ml, DSHB, 70-100-1) and a goat anti-mouse secondary antibody (1:10000, Li-Cor, 926–32210) were used to confirm the presence of porin. A streptavidin conjugate (1:5000, Invitrogen, S21378) which binds to the mitochondrial protein MCCC1 (mitochondrial 3-methylcrotonyl-CoA carboxylase α [34]) was used to measure the levels of this mitochondrial protein. Blots were analysed using Odyssey software. Total protein loaded was stained with Revert 700 Total Protein Stain (Li-Cor, 926–11010) and imaged and quantified on the Odyssey CLx.

### 2.4 Mitochondrial respirometry function

The effects that REMIT expression may have on mitochondrial stress were investigated in real time [35]. In these experiments, a Seahorse XF^e24^ Extracellular Flux Analyzer was used to measure mitochondrial respirometry within REMIT expressing cells, wildtype cells and cells expressing mRFPmars lacking the mitochondrial localization sequence. Mitochondrial respirometry was measured in terms of the basal O_2_ consumption rate, the O_2_ consumption rate devoted to the synthesis of ATP, the maximum O_2_ consumption rate, the contribution of Complex I to the maximum O_2_ consumption rate, the contribution of Complex II to the maximum O_2_ consumption rate, and the O_2_ consumption rate devoted to mitochondrial function other than ATP synthesis, i.e. “proton leak”.

### 2.5 Statistical analysis

The distribution of all experimental data was tested using the Anderson-Darling test for normality. All data that showed a Gaussian distribution were analysed using parametric tests. Data from two groups not showing a Gaussian distribution were analysed using a Mann-Whitney T-test. The one-way analysis of variance (ANOVA) statistical test was used to test for significance between the means of three or more independent groups of normally distributed data.

## 3 Results and discussion

**Fig. 2:**
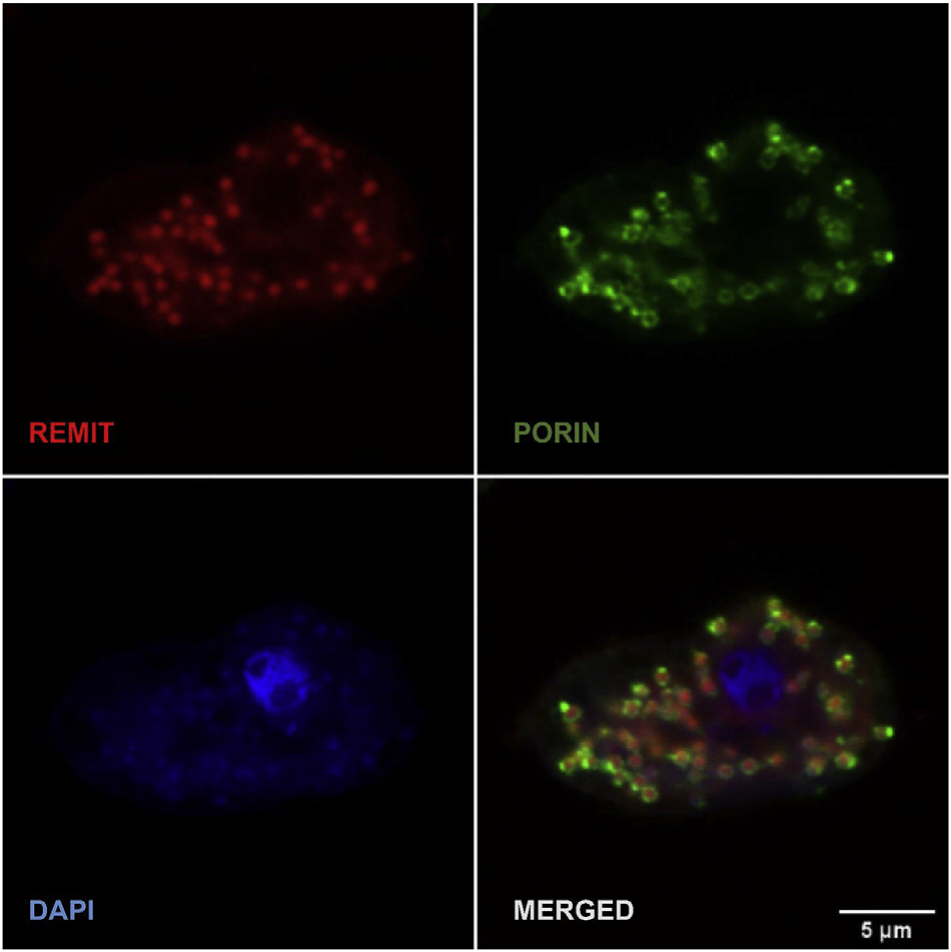
**Fixed-cell imaging of REMIT localizing to the mitochondria**. (A) *D. discoideum* cells expressing REMIT-mRFPmars (red), were fixed and immunolabelled using an anti-porin antibody (green), and stained with DAPI to visualize DNA (blue). Confocal single plane imaging showed red fluorescent protein localizing to mitochondria in *D. discoideum*. Scale bar correspond to 5 μm. (For interpretation of the references to colour in this figure legend, the reader is referred to the Web version of this article.)

**Fig. 3:**
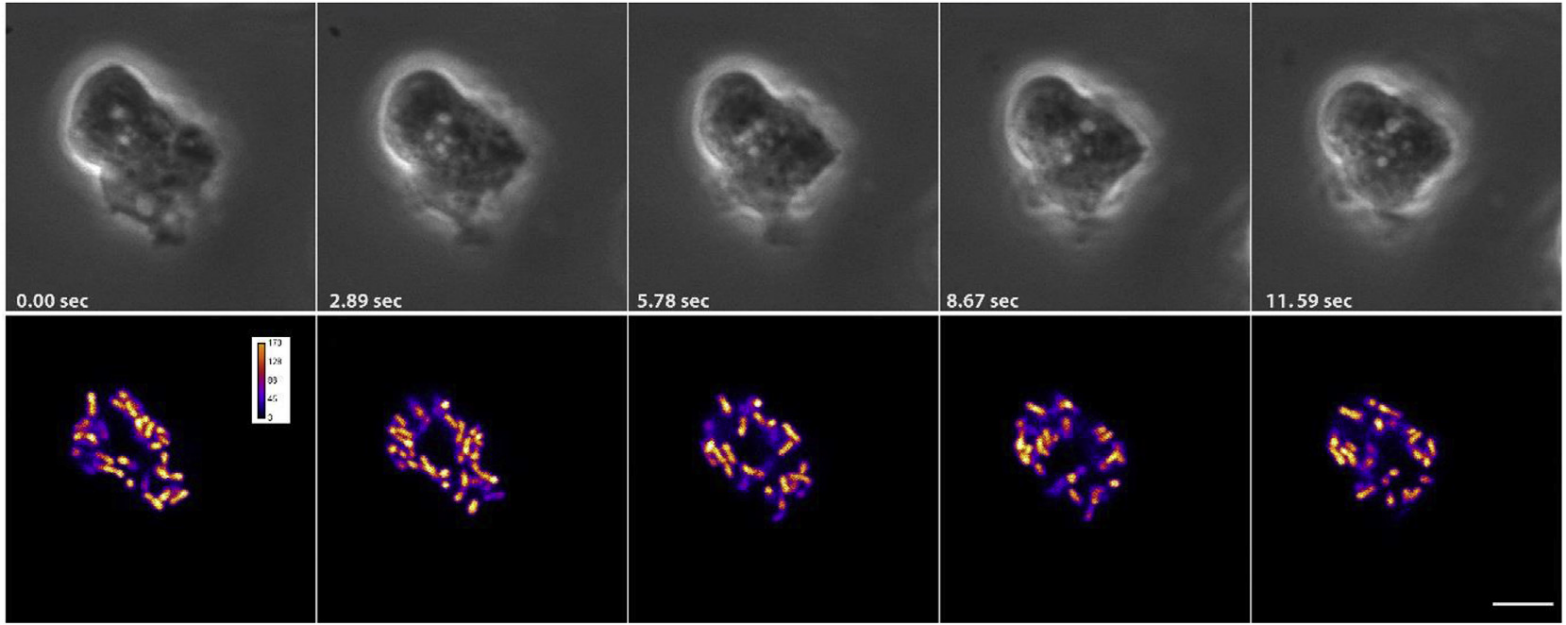
**Live-cell imaging of REMIT localizing to the mitochondria**. (A) Time lapse single plane imaging on a confocal microscope showing *D. discoideum* cells transfected with REMIT-mRFPmars. The upper panel shows phase contrast images, the lower panels the intensity of the mRFPmars signal according to grey levels depicted in colour-mode fire [38], scale bar 5 μm. Images correspond to Supplementary movie 1. Similar live cell imaging using red fluorescence are provided in Supplementary movie 2, using under agar inverted fluorescence in Supplementary movie 3, Z stack imaging in Supplementary movie 4, and 3D reconstruction of live-cell imaging in Supplementary movie 5. (For interpretation of the references to colour in this figure legend, the reader is referred to the Web version of this article.)

The *D. discoideum* mitochondrial localization sequence (MLS) (Fig. 1) was derived from the GcvH1 protein, a member of the mitochondrial glycine cleavage system enzyme complex [26]. The glycine cleavage system is located within the mitochondrial matrix and has a loose affiliation with the inner mitochondrial membrane. We cloned the GcvH1 MLS onto the N-terminus of mRFPmars [27] to form the REMIT vector that was then transfected into wildtype *D. discoideum* cells. To validate the mitochondrial localization of mRFPmars in these cells, mRFPmars fluorescence was examined in fixed cells [36] (Fig. 2), showing highly localised distribution in mitochondrial-like structures. Since porin is localised in the outer mitochondrial membrane, we examined this localization, indicating REMIT mRFPmars in the mitochondrial matrix with porin surrounding this labelling (Fig. 2). We then assessed the use of REMIT mRFPmars for labelling mitochondria in live cell imaging experiments. By using live-cell confocal microscopy, REMIT-expressing cells revealed a highly discrete labelling of mitochondria in real time (Fig. 3, and Supplementary movies 1,2), also visible using under-agar inverted fluorescence microscopy (Supplementary movie 3). A similar localization was found using a GFP-encoding vector, GREMIT (Supplementary Fig. 1). We also employed high resolution live-cell imaging to monitor mitochondrial dynamics (Supplementary movie 4), and this enabled 3-dimensional reconstruction of mitochondrial distribution in live cells (Supplementary movie 5).

Supplementary video related to this article can be found at https://doi.org/10.1016/j.bbrep.2020.100751

The following are the supplementary data related to this article:

Supplementary Movie 1 Confocal single plane imaging of live REMIT-expressing wild-type *Dictyostelium* cells, with intensity indicated by colour. Imaging corresponds to that shown in Fig. 3, and the frame rate is 5 images per second. Scale bar, 5 μm. Supplementary Movie 1

Supplementary Movie 2 Confocal single plane imaging of live REMIT-expressing wild-type *Dictyostelium* cells, showing red flourescence. Imaging corresponds to that shown in Fig. 3, and the frame rate is 5 images per second. Scale bar, 5 μm. Supplementary Movie 2

Supplementary Movie 3 REMIT-expressing WT *Dictyostelium cells*, imaged with live cells using inverted fluorescence microscopy under agar (Olympus IX71). The frame rate is 1 image per second. Scale bar correspond to 5 μm. Supplementary Movie 3

Supplementary Movie 4 Z-stacks of live REMIT-expressing WT *Dictyostelium cells* were imaged by high-resolution microscopy (Zeiss LSM 900 Airyscan). Imaging is recorded at the frame rate of 5 images per second. Supplementary Movie 4

Supplementary Movie 5 3D reconstruction of a z-scan recorded by high-resolution microscopy (Zeiss LSM 900 Airyscan 2) of live REMIT-expressing WT *Dictyostelium* cells. The first stack from the data set of Supplementary Movie 4 was used for the 3D reconstruction. Supplementary Movie 5

**Fig. 4:**
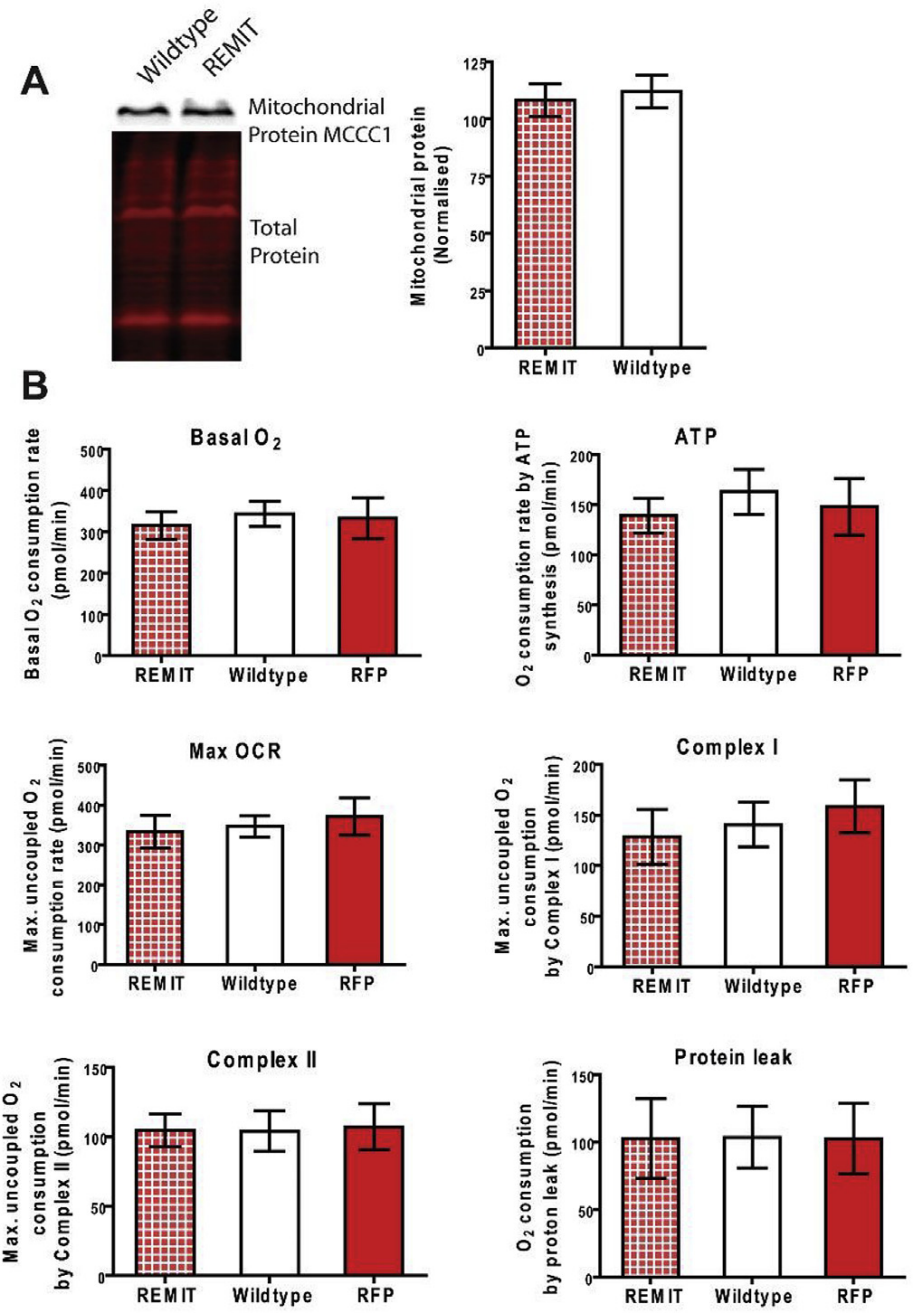
**Expression of REMIT within *D. discoideum* has no effect on mitochondrial load or respiratory function**. Mitochondrial load and respiratory function was evaluated in *D. discoideum* cells transfected with the REMIT plasmid. (A) Western blot analysis was used to assess levels of a mitochondrial-specific marker protein (MCCC1) and total protein levels. Data represent the mean and SEM (P > 0.05), n = 6 (Mann-WhitneyU test). (B) Mitochondrial respiratory function was evaluated in cells transfected with REMIT, with wildtype cells, and with the REMIT plasmid lacking the localization sequence (RFP). Respiratory function was quantified in terms of basal O_2_ consumption rate, O_2_ consumption rate devoted to the synthesis of ATP, maximum O_2_ consumption rate, contribution of complex I to the maximum O_2_ consumption rate, contribution of complex II to the maximum O_2_ consumption rate, O_2_ consumption rate devoted to mitochondrial function other than ATP synthesis, i.e. “proton leak”. Data represent the mean and SEM (P > 0.05), n = 6 (One way ANOVA).

In order to maintain a healthy population of mitochondria within the cells, all mitochondria experiencing damage or dysfunction will undergo mitophagy. This process results in defective mitochondria being targeted to the lysosome for autophagic degradation, thereby maintaining cell health [37]. Because transfection with REMIT leads to the localization of mRFPmars to the mitochondria we investigated whether this resulted in mitochondrial damage or dysfunction, resulting in mitophagy, or alternatively increased mitochondrial load (the mitochondrial protein content). To assess mitochondrial loading, we compared levels of a mitochondrial protein, mitochondrial 3-methylcrotonyl-CoA carboxylase α [34], in wildtype cells and cells transfected with REMIT using Western blot analysis (Fig. 4A). From this analysis, no change in mitochondrial load was identified following REMIT transfection.

The requirement of mitochondria to carry out normal respiratory function is fundamental to maintaining a healthy cell. Any deleterious effect on respiratory function as a result of REMIT transfection would result in downstream processes being disrupted. Thus, it is necessary to confirm that mitochondrial respiratory function is not disrupted. Mitochondrial respirometry function was therefore measured in terms of the basal O_2_ consumption rate, the O_2_ consumption rate devoted to the synthesis of ATP, the maximum O_2_ consumption rate, the contribution of complex I to the maximum O_2_ consumption rate, the contribution of complex II to the maximum O_2_ consumption rate, and the O_2_ consumption rate devoted to mitochondrial function other than ATP synthesis, i.e. “proton leak” (Fig. 4B). These data were obtained from cells transfected with REMIT, cells transfected with REMIT lacking the MLS, and untransfected wildtype cells. For all six conditions no significant difference (P > 0.05) was found between the three cell lines. This shows that mitochondrial function is not affected despite the presence of mRFPmars localised within the mitochondrial matrix.

These experiments thus show that transfection of *D. discoideum* cells with REMIT provides a quick, cheap and convenient method to visualize mitochondria in real time. The use of REMIT would be advantageous in studies involving the need for both visualization and normal respiratory function. These studies can include investigation into mitochondrial fission and fusion events, mitochondrial dynamics and mitochondrial morphology.

## Author statement

CJP and RSBW conceived the research and wrote the paper. ECW and JD-O contributed to imaging and measuring mitochondria load. CS, LMF, SJA, PRF analysed mitochondrial function. AM-T provided confocal microscopy and 3D modelling. All authors contributed to editing the paper.

## Declaration of competing interest

The authors declare that they have no known competing financial interests or personal relationships that could have appeared to influence the work reported in this paper.

## Appendix A Supplementary data

Supplementary Fig. 1 GREMIT-expressing WT *Dictyostelium cells*, imaged with live cells using inverted fluorescence microscopy under agar (Olympus IX71). Scale bar correspond to 5 μm. Supplementary Fig. 1
