# Supplementary material for: A Dictyostelium discoideum mitochondrial fluorescent tagging vector that does not affect respiratory function
Source: Biochem Biophys Rep. 2020 Mar 25;22:100751. doi: 10.1016/j.bbrep.2020.100751 (PMC7109396; doi:10.1016/j.bbrep.2020.100751)
Supplement: Supplementary Fig. 1 — GREMIT-expressing WT Dictyostelium cells, imaged with live cells using inverted fluorescence microscopy under agar (Olympus IX71). Scale bar correspond to 5 μm. [file mmc6.docx]

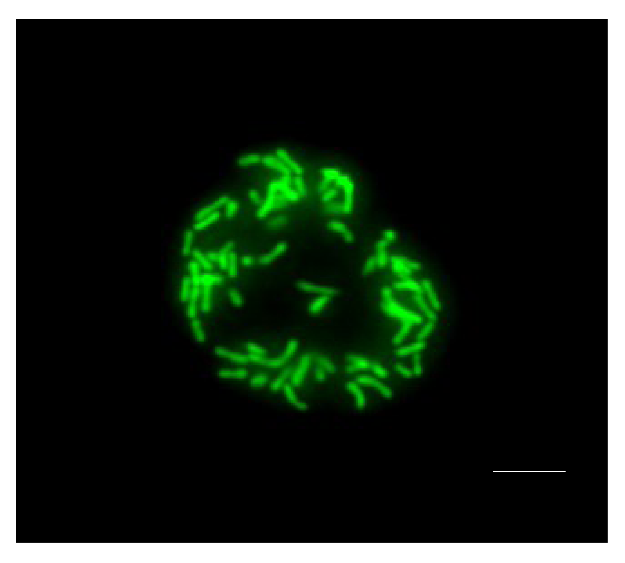


**Supplementary Figure 1.** GREMIT-expressing WT *Dictyostelium cells*, imaged with live cells using inverted fluorescence microscopy under agar (Olympus IX71). The frame rate is 1 images per second. Scale bar correspond to 5 µm.
